# Host markers in Quantiferon supernatants differentiate active TB from latent TB infection: preliminary report

**DOI:** 10.1186/1471-2466-9-21

**Published:** 2009-05-16

**Authors:** Novel N Chegou, Gillian F Black, Martin Kidd, Paul D van Helden, Gerhard Walzl

**Affiliations:** 1 Division of Molecular Biology and Human Genetics, Department of Biomedical Sciences, Faculty of Health Sciences, University of Stellenbosch, Cape Town, Western Cape Province, P.O. Box 19063, Tygerberg 7505, South Africa; 2 Centre for Statistical Consultation, Department of Statistics and Actuarial Sciences, Faculty of Science, University of Stellenbosch, Cape Town, Western Cape Province, Private Bag X1, Matieland 7602, South Africa

## Abstract

**Background:**

Interferon gamma release assays, including the QuantiFERON^® ^TB Gold In Tube (QFT) have been shown to be accurate in diagnosing *Mycobacterium tuberculosis *infection. These assays however, do not discriminate between latent TB infection (LTBI) and active TB disease.

**Methods:**

We recruited twenty-three pulmonary TB patients and 34 household contacts from Cape Town, South Africa and performed the QFT test. To investigate the ability of new host markers to differentiate between LTBI and active TB, levels of 29 biomarkers in QFT supernatants were evaluated using a Luminex multiplex cytokine assay.

**Results:**

Eight out of 29 biomarkers distinguished active TB from LTBI in a pilot study. Baseline levels of epidermal growth factor (EGF) soluble CD40 ligand (sCD40L), antigen stimulated levels of EGF, and the background corrected antigen stimulated levels of EGF and macrophage inflammatory protein (MIP)-1β were the most informative single markers for differentiation between TB disease and LTBI, with AUCs of 0.88, 0.84, 0.87, 0.90 and 0.79 respectively. The combination of EGF and MIP-1β predicted 96% of active TB cases and 92% of LTBIs. Combinations between EGF, sCD40L, VEGF, TGF-α and IL-1α also showed potential to differentiate between TB infection states. EGF, VEGF, TGF-α and sCD40L levels were higher in TB patients.

**Conclusion:**

These preliminary data suggest that active TB may be accurately differentiated from LTBI utilizing adaptations of the commercial QFT test that includes measurement of EGF, sCD40L, MIP-1β, VEGF, TGF-α or IL-1α in supernatants from QFT assays. This approach holds promise for development as a rapid diagnostic test for active TB.

## Background

Commercial *in vitro *T-cell interferon gamma (IFN-γ) release assays (IGRAs) including the QuantiFERON^® ^tests (Cellestis, Victoria, Australia) and T SPOT. *TB *(Oxford Immunotec, Abington, UK) have been introduced into clinical practice for the diagnosis of *Mycobacterium tuberculosis *(*M. tb*) infection. These assays make use of *M. tb *specific antigens, ESAT-6 and CFP-10, and a third antigen, TB7.7 (Rv2654) in the QuantiFERON^® ^TB Gold In-Tube (QFT).

The IGRAs (*reviewed in *[1]) employ whole blood or peripheral blood mono nuclear cells, which are cultured overnight with the TB specific antigens. *M. tb *infected individuals harbour pre-activated T-cells which rapidly respond by the release of cytokines including IFN-γ when challenged with *M. tb *antigens. The IFN-γ released by these activated cells is then quantitated by ELISA in the QuantiFERON assays or by enumeration of spot-forming cells in the ELISPOT-based T SPOT. *TB *[1].

IGRAs have been extensively studied, and shown to be very sensitive and specific for latent *M. tb *infection (LTBI) especially in comparison to the tuberculin skin test (TST) [2-5]. The many other advantages offered by these assays over the TST have been well documented [1,3,6].

The current standard tests for active tuberculosis (TB) have serious limitations. Sputum smear examination for acid-fast bacilli (AFB) has a low sensitivity and cannot discriminate between *M. tb *and non tuberculous mycobacteria, and sputum culture for *M. tb *takes several days to weeks to yield a result [7]. Diagnosing TB in sputum smear and culture-negative patients and in those with extra-pulmonary disease remains challenging [8]. While IGRAs are useful in the diagnosis of *M. tb *infection, an important limitation of these assays is their inability to discriminate between LTBI and active TB. These assays are therefore of little value in high TB incidence areas with a very high LTBI burden. Discovery of biomarkers that can rapidly differentiate between the two infection states would be a major breakthrough.

Recent technological advances have made it possible to screen for many biomarkers in as little as 25 μl of sample using Luminex multiplex cytokine beaded arrays. We hypothesized that *M. tb *specific antigenic stimulation of whole blood would result in the production of multiple biomarkers, some of which would be unique to either LTBI or active TB disease. In the present study, levels of 29 markers are measured in QFT supernatants and promising analytes are identified with ability to discriminate between LTBI and active TB.

## Methods

### Study subjects

We sequentially recruited 23 pulmonary TB patients and 34 household contacts (HHC) of pulmonary TB patients from the Ravensmead/Uitsig community in the Western Cape Province of South Africa between October 2006 and April 2007. The TB incidence in South Africa was 940 per 100,000 while the case notification rate was 628 per 100,000 in 2006[9]. BCG vaccination (Danish strain, 1331, Statens Serum Institute, Copenhagen, Denmark) is routinely administered at birth in the study area. All the pulmonary TB patients were self-reporting, untreated cases with a first episode of TB and were all AFB positive on two smears. HHCs had been living in the same house as an adult TB case who was diagnosed not more than 2 months before recruitment of the contact. All HHCs had normal chest X-rays and AFB negative assisted sputum samples. Inclusion criteria for all participants were: age 10 to 60 years, negative HIV test (Abbot Determine™ HIV 1/2; Abbott, Wiesbaden, Germany), willingness to give written informed consent for participation and availability for TST reading at 48–72 hours (HHCs only). Exclusion criteria for participants included previous or current TB treatment, serious concomitant chronic conditions, steroid therapy within the past 6 months and pregnancy. Demographic data was collected and a clinical questionnaire completed. Ethical approval for the study was obtained from the Committee for Human Research of the University of Stellenbosch.

### Diagnostic tests

At enrolment, 10 ml of heparinized blood was collected from all participants and transported (at ambient conditions) within 2 hours of collection to the laboratory. The QFT test (using 3 ml of blood) was performed on all study subjects and interpreted for TB infection according to the manufacturer's instructions [10] (see details below). The TST, using 2 TU PPD (Mantoux PPD, Statens Serum Institute), was performed on all HHC after blood collection.

### IFN-γ measurement and initial screening for biomarkers

IFN-γ measurement in QFT supernatants was done with the QFT ELISA [10]. Tests were regarded as positive for TB infection if the difference between the TB antigen (stimulated) and the unstimulated supernatant was = 0.35 IU/ml regardless of mitogen value. The tests were judged as negative when this difference was < 0.35 IU/ml, provided that the value of mitogen stimulated supernatant was ≥ 0.5 IU/ml after subtraction of the unstimulated value. These results were generated using the QFT analysis software, version 2.50.

To reduce cost we initially performed 29-plex Luminex assays on a subset of randomly selected supernatants from 10 TB cases and 9 HHCs with positive QFT results. We only included QFT positive individuals in the pilot study because our objective was to identify markers that could discriminate between LTBI and active TB. Levels of interleukin (IL)-1α, IL-1β, IL-2, IL-4, IL-5, IL-6, IL-7, IL-10, IL-12(p40), IL-12(p70), IL-13, IL-15, IL-17, IL-8 (CXCL8) interleukin 1 receptor antagonist (IL-1ra), soluble CD40 ligand (sCD40L), epidermal growth factor, (EGF), eotaxin (CCL11), fractalkine, granulocyte colony stimulating factor (G-CSF), granulocyte monocyte stimulating factor (GM-CSF), interferon gamma (IFN-γ), interferon inducible protein 10 (IP-10 or CXCL10), monocyte chemotactic protein-1 (MCP-1 or CCL2), macrophage inflammatory protein (MIP)-1α or CCL3, MIP-1β (CCL4), transforming growth factor (TGF)-α, tumour necrosis factor (TNF)-α and vascular endothelial growth factor (VEGF) were evaluated in unstimulated (cytokine_Nil_), *M. tb *specific antigen (cytokine_Ag_) and phytohaemagglutinin (mitogen) stimulated QFT supernatants.

We used the unstimulated (Nil), *M. tb *antigen stimulated (Ag) and mitogen stimulated supernatant data, as well as the difference between the antigen stimulated (Ag-Nil) or the mitogen stimulated (Mit-Nil) and the unstimulated supernatant levels as separate variables in analysis of the data. This was done to allow evaluation of baseline marker levels, *M. tb *antigen or mitogen stimulated levels and differences between these levels in differentiating between TB infection states.

The performance of single and sets of biomarkers in differentiating between active TB and absence of active TB was evaluated in a) QFT positive samples, and b) all samples regardless of QFT result.

Eight out of 29 biomarkers that showed significant differences or trends for differences between LTBI and active TB after evaluation on the 19 QFT positive subjects were selected and evaluated on the rest of the participants (n = 38) with a customized 8-plex kit. The 8 markers were IL-1α, sCD40L, EGF, IFN-γ, MIP-1β, TGF-α, TNF-α and VEGF. The data collected on these 8 analytes from the 19 participants tested with the 29-plex kit was combined with the data collected on the remaining 38 participants tested with the customized 8-plex kit for the final analysis.

### Luminex assay

Biomarker levels were measured using LINCO-plex^® ^kits (Millipore, St. Charles, Missouri, USA) on the Bio Plex platform (Bio Plex™, Bio Rad Laboratories) according to the Linco instructions [11]. All supernatants were diluted 1:1 with the kit serum matrix diluent, following optimization experiments. Only the unstimulated and *M. tb *antigen stimulated supernatants were used in the customized 8-pex kits as the levels of markers in the mitogen stimulated supernatants evaluated with the 29-plex were not useful in the models for differentiating between LTBI and active TB. All samples were evaluated in duplicate by a single technician who was blinded to participant groups. All analyte levels in the quality control reagents included in the kits were within the expected ranges. To access the variability in sample runs, a supernatant from a single QFT positive household contact (R386) was evaluated on all plates. Both the intra-plate and inter-plate coefficients of variation for duplicate runs of this sample varied between analytes, but were mostly below 20% (range, 9.5% – 41.3%). The standard curve for all biomarkers ranged from 3.2–10000 pg/ml. Bio-Plex Manager Software, version 4.1.1 was used for the analysis of the data.

### Statistical analysis

IFN-γ levels measured by the QFT ELISA (IU/ml) were converted to pg/ml by multiplying by a factor of 40 [12]. All analyte levels obtained with the Luminex assay were multiplied by 2 to correct for the dilution. Differences between study groups were determined using the Mann-Whitney U test. Cut-off levels for differentiating between groups were determined by receiver operator characteristic (ROC) curve analysis using the "R" statistical programming language. General discriminant analysis (GDA) and support vector machine (SVM) models (described in [13]) were used to evaluate the predictive abilities of combinations of biomarkers for differentiating between *M. tb *infection states. Optimal combinations of biomarkers were investigated by performing best subsets analysis in both cases (GDA and SVM). Prediction accuracy were estimated using leave-one-out cross validation. This method was used due to the small sample size. A 5% significance level was used as guideline for determining significant associations. The data was analysed using the Statistica 8 software, Statsoft (Ohio, USA).

## Results

### QFT testing

All the household contacts with a positive QFT test (73.5%) also had a positive TST (10 mm cut-off). Only 10 out of the 57 participants evaluated in the study had negative QFT tests and no indeterminate results were observed. The demographic and clinical information collected on the participants is shown in table 1.

**Table 1:** Clinical and demographic characteristics of study subjects.

	**All**	**Pulmonary TB**	**Household contacts**
**Number of participants**	57	23	34
**Age, mean yr ± SD**	31.2 ± 13.9	30.3 ± 13.6	31.8 ± 14.2
**Age range, yr**	10.1 – 59.9	10.1 – 57.4	10.7 – 59.9
**Male/female ratio**	31/26	17/6	14/20
**TST mean, mm**	na	nd	22.8
**TST range, mm**	na	na	0.0–46.0
TST pos*, %	na	na	87.5
**QFT pos, %**	82.5	95.6	73.5

QFT = QuantiFERON TB Gold In Tube, TB = active tuberculosis, TST = tuberculin skin test, yr = years, SD = standard deviation, na = not applicable, nd = not done, * = Two household contacts did not return for TST reading.

### Analysis of QFT supernatants with the 29-plex kit and selection of promising markers for customized 8-plex kit

*M. tb *antigen stimulation of whole blood resulted in the production of significantly higher levels of sCD40L and VEGF in latently infected individuals compared to active TB patients. There were also significant differences in the unstimulated levels of EGF, TGF-α, TNF-α and sCD40L between LTBI and active TB (Table 2). IL-1α, MIP-1β and IFN-γ showed borderline differences between the two groups and were included in the customized 8-plex kit. An excellent correlation was observed between the ELISA and Luminex measured IFN-γ levels (both with the 29- and 8-plex assays), although the levels measured by ELISA were often higher than those detected by the Luminex assay (r = 0.88; p < 0.0001).

**Table 2:** Median levels of promising analytes as measured with the 29-plex assay

Analyte	All subjects **(n = 19)**	Active TB cases **(n = 10)**	LTBI **(n = 9)**	p-value (Active TB vs LTBI)
**EGF**_**Nil**_	73.0 (23.4–148.9)	106.2 (40.8–148.9	51.4 (23.4–141.3	0.02
**IFN-γ**_**Ag-Nil**_	99.1 (0.0–1265.0)	57.0 (0.0–646.3)	127.6 (38.6–1265.5)	**0.08**
IP-10* _**Ag-Nil**_	18139.0 (5313.0–>20000.0)	14446.0 (5313.0–>20000.0)	18224.0 (12470.0–>20000.0)	**0.27**
IL-2*_Ag-Nil_	160.0 (10.2–1778.0)	106.6 (10.2–640.9)	164.0 (57.8–1778.0)	**0.19**
**IL-1α**_**Ag-Nil**_	76.6 (0.0–235.4)	118.0 (0.0–235.4)	23.3 (0.0–158.5)	0.04
**MIP-1β**_**Ag-Nil**_	1770 (0.0–7847.0)	1210.0 (0.0–4098.0)	3220 (960.8–7847.0)	**0.06**
**TNF-α**_**Nil**_	50.8 (7.3–633.7)	94.9 (19.8–633.7)	15.9 (7.3–293.6)	0.01
**TGF-α**_**Nil**_	13.3 (0.0–230.1)	28.5 (0.0–230.1)	6.2 (0.0–13.3)	<0.01
**VEGF**_**Ag-Nil**_	0.0 (0.0–77.3)	0.0 (0.0–31.8)	36.3 (0.0–77.3)	<0.01
**sCD40L**_**Nil**_	869.0 (245.2–9852.0)	1388.0 (455.4–4038.0)	599.4 (245.2–9852.0)	0.03
**sCD40L**_**Ag-Nil**_	0.0 (0.0–9944.0)	0.0 (0.0–4.6)	309.4 (12.2–9944.0)	<0.01

Median levels (pg/ml) and ranges (in parenthesis) of analytes with significant or near significant differences between TB cases and the latently infected individuals obtained with the 29-plex assay. * Marker was not included in the customized 8-plex kit. Levels shown as >20000 pg/ml were above the highest point on the standard curve.

### Ability of eight selected markers to diagnose active TB

#### a) Discrimination between LTBI and TB disease in QFT positive supernatants

**Figure 1:**
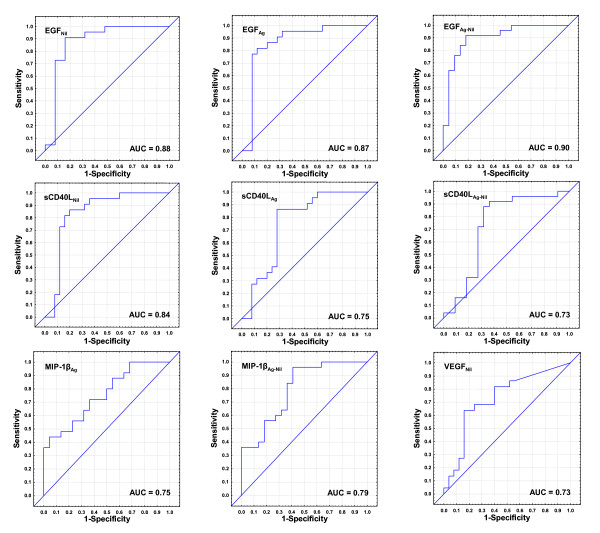
**Receiver operator characteristics curves showing the accuracies of top individual analytes in discriminating between active TB and latent TB infection**. Receiver operator characteristic (ROC) curves for the accuracies of single analytes to differentiate between active TB and LTBI in QFT positive individuals. Only ROC curves for markers that differentiated between the two infection states with AUCs ≥ 0.73 are shown. AUC = Area under the curve.

Unstimulated (_Nil_), TB antigen stimulated (_Ag_) and antigen stimulated minus unstimulated (_Ag-Nil_) levels of EGF, sCD40L, and TGF-α_Ag_, MIP-1β_Ag-Nil _and VEGF_Nil _were the most accurate single markers that differentiated between the two infection states. The median levels of the individual markers in the two groups, cut-off values and their respective accuracies (sensitivity and specificity) in distinguishing QFT positive pulmonary TB cases from QFT positive HHCs are shown in table 3 while ROC curves are shown in figure 1.

**Table 3:** Abilities of top individual analytes to discriminate positive QFT results as active TB or LTBI

Marker	Level in Pulmonary TB (n = 22)	Level in LTBI (n = 25)	Cut-off (pg/ml)	Sensitivity %	Specificity %	PPV, %	NPV, %
**EGF**_**Nil**_	115.5 (39.1–222.2)	34.5 (15.2–214.0)	76.16	90.9	84.0	83.3	91.3
**EGF**_**Ag**_	65.7 (23.4–121.8)	27.2 (12.2–487.9)	46.68	81.8	88.0	85.7	84.6
**EGF**_**Ag-Nil**_	-44.9 (-116.1–0.44)	-7.0 (-48.9–274.0)	-25.6	92.0	81.8	85.1	90.0
**TGF-α**_**Ag**_	22.2 (1.0–168.0)	13.4 (4.3–47.1)	13.78	81.8	60.0	64.3	78.9
**IFN-γ**_**Ag-Nil**_	99.3 (-216.4–699.8)	206.9 (8.7–6112.0)	106.68	80.0	54.5	66.7	70.6
**sCD40L**_**Nil**_	3995.0 (876.1–10245.0)	1002.0 (220.4–>20000)	2307.54	86.4	80.0	79.2	87.0
**sCD40L**_**Ag**_	1974.0 (883.0–13493.0)	1040.0 (383.2–>20000)	1563.66	86.4	72.0	73.1	85.7
**sCD40L**_**Ag-Nil**_	-1502.0 (-5339.0–3496.0)	10.6 (-3527.0–3927.0)	-471.06	88.0	68.2	75.9	83.3
**MIP-1β**_**Ag-Nil**_	458.5 (-1035–2321.0)	1833.0 (290.0–6890.0)	836.18	84.0	63.6	72.4	77.8
**VEGF**_**Nil**_	83.4 (0.0–482.3)	19.5 (0.0–415.8)	72.78	63.6	84.0	77.8	72.4

Median levels (pg/ml) and ranges (in parenthesis) of individual markers measured in QFT positive supernatants and abilities to discriminate between positive QFT results as either pulmonary TB or LTBI. Only markers with either sensitivity and/or specificity ≥ 80.0% between groups are shown. LTBI = household contacts with both positive QFT and TST results, PPV = positive predictive value, NPV = negative predictive. Levels shown as >20000 pg/ml were above the range of the standard curve.

**Figure 2:**
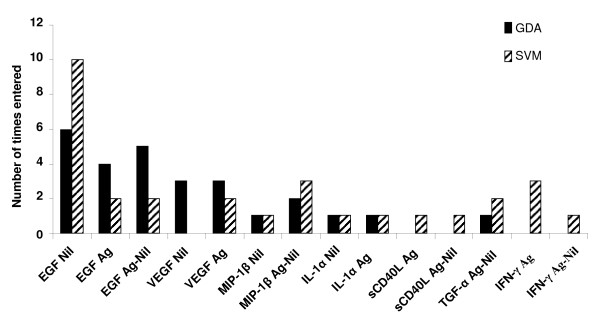
**Frequency of individual analytes in top models for discriminating between active TB and latent TB**. The columns represent the number of inclusions of individual markers into the most accurate three-analyte models by general discriminant and support vector machine analysis (6 and 10 models, respectively) for discriminating between active pulmonary TB cases and LTBI in participants with positive QFT results.

Fitting two mathematical models (general discriminant analysis [GDA] and support vector machines [SVM]) to the data indicated that optimal prediction of TB infection states could be achieved with combinations of 3 variables. EGF_Nil _was the most frequently occurring marker in both the GDA and SVM biomarker combinations differentiating between the QFT positive pulmonary TB cases and the QFT positive HHCs (figure 2). A combination of EGF_Nil_, MIP-1β_Ag-Nil _and IL-1α_Nil _(or IL-1α_Ag_) classified pulmonary TB cases with an accuracy of 95.5% in a resubstitution classification matrix and with 90.9% after leave-one-out cross validation. The same biomarker combination classified the QFT positive HHCs with an accuracy of 88.8% after leave-one-out cross validation. Other three-variable combinations including any two of EGF_Nil_, EGF_Ag _or EGF_Ag-Nil _plus a third marker selected from VEGF_Nil_, VEGF_Ag_, TGF-α_Ag-Nil _or MIP-1β_Nil _in GDA, classified the QFT positive TB cases with accuracies above 90.0% in resubstitution classification matrix (range, 90.9–95.5%) and above 86.0% (range, 86.4–90.9%) after leave-one-out cross validation. The same combinations of analytes accurately classified more than 88.0% of HHC (range, 88.8–96.0%) in resubstitution classification matrix and up to 88.0% (range, 84.0–88.0%) after leave-one-out cross validation.

In SVM analysis, two three-marker combinations (EGF_Nil_/EGF_Ag-Nil_/MIP-1β_Ag-Nil _and EGF_Nil_/IL-1α_Nil_/MIP-1β_Ag-Nil_) differentiated QFT positive TB cases from QFT positive HHCs with overall accuracies of 86.0% and 90.4% respectively, and above 85.0% after leave-one-out cross validation. The predictive abilities of the top 6 and 9 three-marker combinations in GDA and SVM models, for differentiating between positive QFT results as active TB or LTBI, are shown on additional files 1 and 2 respectively.

#### b)i. Differentiating between TB cases and household contacts irrespective of QFT results

**Figure 3:**
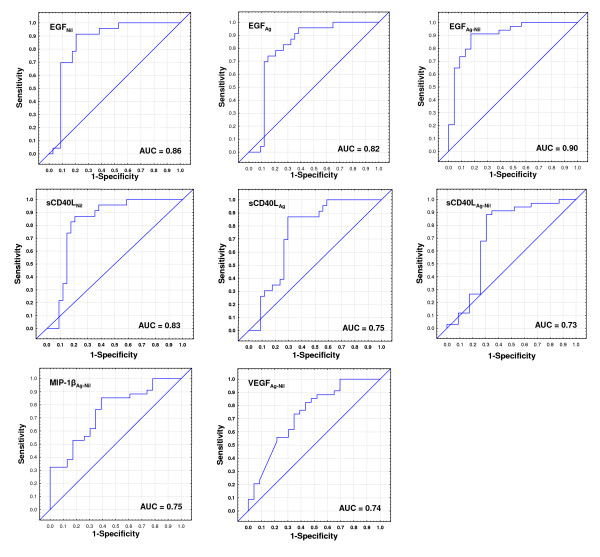
**Receiver operator characteristics curves showing the accuracies of top individual analytes in discriminating between TB disease and the absence of active TB irrespective of QFT results**. Only ROC curves for markers that differentiated between groups with AUCs ≥ 0.73 are shown. AUC = Area under the curve.

**Figure 4:**
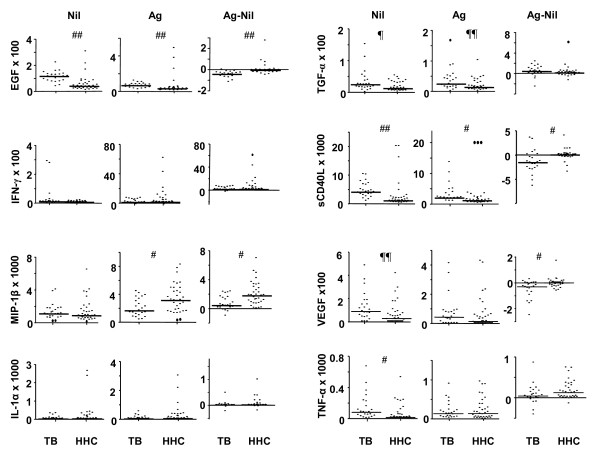
**Levels of individual analytes in all TB cases (TB) and household contacts (HHC)**. Each dot represents the analyte level of one participant in the study and horizontal lines represent the median values. Asterixes indicate significant differences between the TB cases (n = 23) and household contacts (n = 34). ##: p < 0.0001, #: p < 0.01, ¶¶: p = 0.01, ¶: p = 0.02. Nil: unstimulated analyte levels, Ag: Levels obtained after stimulation with *Mycobacterium tuberculosis *specific antigen cocktail (ESAT-6, CFP-10 and TB7.7), Ag-Nil: difference between the *Mycobacterium tuberculosis *specific antigen stimulated and the unstimulated levels.

EGF_Nil/Ag-Nil_, sCD40L_Nil/Ag/Ag-Nil_, MIP-1β_Ag-Nil _and TGF-α_Ag _were the most accurate single markers that differentiated between pulmonary TB cases and HHCs irrespective of QFT results (figures 3 and 4).

**Figure 5:**
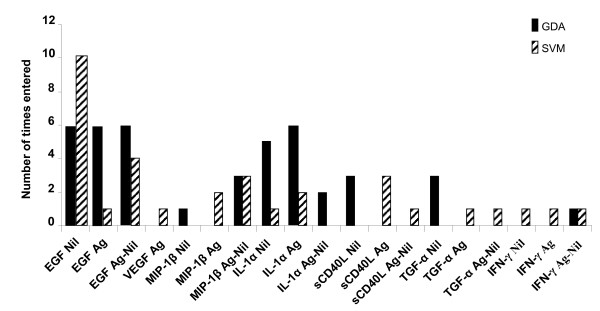
**Frequency of individual analytes in models for discriminating between active TB and no active TB**. The columns represent the number of inclusions of individual markers into the most accurate three-analyte models by general discriminant and support vector machine analysis (6 and 10 models, respectively) in discriminating between active pulmonary TB cases and participants without active TB irrespective of QFT results.

EGF_Ag-Nil _was the most frequently occurring marker in the top three-analyte GDA model combinations that most accurately differentiated cases from contacts irrespective of QFT results, while EGF_Nil _and EGF_Ag-Nil _were the most frequent in the top SVM model combinations (figure 5).

Three-marker models comprising i) EGF_Nil_, EGF_Ag _or EGF_Ag-Nil_, or ii) any two of the EGF conditions plus any one of IL-1α_Nil_, IL-1α_Ag _or MIP-1β_Ag-Nil_, or iii) any one of the EGF conditions plus any two of IL-1α_Nil_, IL-1α_Ag _or MIP-1β_Ag-Nil _differentiated between TB cases and HHCs in GDA with accuracies up to 96.0% (range, 87.0–96.0%) for TB cases and up to 94.1% (range, 85.3–94.1%) for HHCs. In leave-one-out cross validation the accuracies of the biomarker combinations were between 82.6% and 87.0% in TB cases and 85.3% and 91.2% in HHCs.

The top two marker combinations in SVM analysis were EGF_Nil_/EGF_Ag-Nil_/MIP-1β_Ag-Nil _and EGF_Nil_/EGF_Ag-Nil_/IL-1α_Ag_. Both marker combinations correctly classified 87.0% of TB patients and 91.2% of HHCs respectively, with an overall accuracy of 85.3%. The most accurate GDA and SVM model combinations for discriminating between TB cases and HHCs are shown on additional files 3 and 4 respectively.

#### b) ii. Differentiating between QFT positive and QFT negative household contacts

We stratified the household contacts according to QFT status and evaluated whether there were any differences in biomarker levels between them. Of the 8 markers included in the customized kit, only IFN-γ was significantly different between the two groups (TB antigen stimulated levels, p = < 0.001, corrected for background [IFN-γ _Ag-Nil_], p = < 001).

## Discussion

The ability to diagnose TB infection, and distinguish active TB from LTBI by measurement of a limited number of analytes on a small amount of blood in an overnight assay would be a major advance over the currently available TB diagnostic tests. In this study, we have shown for the first time that multiple biomarkers measured in QFT test supernatants have high ability to discriminate between active TB and the absence of active disease. This has significant implications for the diagnostic utility of the QFT test. The top single markers were EGF and sCD40L. Three-marker combinations of EGF with MIP-1β, sCD40L, IL-1α or VEGF showed promising results with the top model comprising EGF_Nil_, EGF_Ag-Nil _and MIP-1β_Ag-Nil_.

The ability of these markers to differentiate between different *M. tb *infection states is probably a reflection of successful and unsuccessful immunological responses against the pathogen. The successful control of *M. tb *infection by the host immune response is largely dependent on T-cells, macrophages and a balance between pro-inflammatory and regulatory cytokines and chemokines. Pulmonary TB granulomas, including areas of caseous necrosis, are rich in growth factors such as EGF, TGF-α and VEGF and provide good growth environments for mycobacteria, including *M. tb *[14,15]. In addition to enhancing the growth of mycobacteria within granulomas, Bermudez and co-workers [14] showed that both *M. tb *and *M. avium *express receptors for EGF. VEGF, an angiogenesis mediator, has been associated with disease activity in both pleural TB and TB meningitis [16,17] and levels decline after successful TB treatment [18]. Both the unstimulated and TB antigen stimulated levels of these growth factors were higher in TB patients than in LTBI in this study.

MIP-1β and IL-1 are produced by macrophages. MIP-1β is known to modulate macrophage functions, is an important mediator of chronic inflammatory processes [19,20], and is a potent macrophage, lymphocyte and specifically activated CD4+ lymphocyte chemo-attractant [20]. IL-1 favours a TH1 immune response [21] and has been shown to play an important role in the formation of granulomas [22] along with TNF-α. Although the Mann-Whitney U test showed no significant differences between the unstimulated levels of MIP-1β, and both the unstimulated and *M. tb *antigen stimulated levels of IL-1α in the different TB infection states, multivariate analysis showed that lower levels of both markers were characteristic of active disease.

CD40L, is a costimulatory molecule that is expressed on activated CD4+ T cells and is involved in their activation and development of effector functions [23]. Mizusawa and co-workers [24] reported significantly higher plasma levels of sCD40L in patients with cavitary TB lesions, compared to those without such lesions. The median levels of sCD40L were higher in TB patients in the present study. While there was no significant difference in the median unstimulated and the antigen stimulated levels in the non-diseased group, unstimulated levels were higher than the antigen stimulated levels in TB patients. Because patients in this study were not classified according to the extent of disease on X-ray, future studies will have to investigate the effect of disease severity on test performance.

Indeterminate results have been an issue of concern, and are often reported in IGRA studies. They frequently occur in immonocompromised subjects [25,26] and have also been observed in children under the age of 5 years [26]. Previous reports have highlighted the potential roles of IP-10, IL-2 and MCP-2 alongside IFN-γ in diagnosing *M. tb *infection [27-29]. These studies revealed that combining IFN-γ and IP-10 measurement in QFT supernatants enhances the sensitivity for diagnosing *M. tb *infection and decreases the proportion of indeterminate results [27,28]. We also observed very high levels of IP-10 in our 29-plex measurements, and which correlated with IFN-γ levels (r = 0.51, p = 0.009) but like IFN-γ, IP-10, which helps to amplify IFN-γ responses by its effects on macrophages, does not differentiate between active TB and LTBI. IFN-γ or IP-10 detection could be used in a first step of a test based on *M. tb *antigen stimulated whole blood culture to diagnose (*M. tb*) infection. A second step of the test could be performed if positive IFN-γ/IP-10 results are obtained to measure three-marker combinations of EGF, sCD40L, MIP-1β, VEGF, IL-1α or TGF-α levels by ELISA or multiplex cytokine assays, to differentiate individuals with active TB from LTBI. Future larger studies should evaluate both QFT positive and negative participants to ascertain whether a one-step three-marker test is sufficient to diagnose active TB or whether a two-step strategy consisting of a conventional QFT test followed by a three-marker assay in those with a positive QFT result yields the best results. Either approach may allow diagnosis of TB disease with high accuracy within 24 hours after presentation of a patient at the health care service, with only 3 ml of blood and without the requirement of a second visit by the patient.

The levels of some of the markers investigated in this study (EGF, sCD40L and VEGF) were lower in the TB antigen stimulated than in the unstimulated QFT tubes. The reasons for this difference might relate to the expression kinetics of the different markers after stimulation with the TB antigens. Another explanation could be that markers are consumed due to possible co-expression of soluble or membrane bound receptors after stimulation. The actual mechanism behind this observation is beyond the scope of this small study and may need to be investigated further in future studies. It has been suggested that some heparinized blood collection tubes may contain endotoxin, which may induce cytokine production during subsequent culture. In the present study blood samples were collected in heparinized tubes prior to transfer to the QFT tubes whereas the manufacturers recommend collection directly in the QFT tubes which are endotoxin free. Possible endotoxin contamination, however, would not explain the higher levels of some analytes in unstimulated than in stimulated samples as the blood from each participant would be collected in a single heparinized tube and both samples would be exposed to the same level of contaminants. Furthermore, as the levels in unstimulated samples were generally not very high, it is unlikely that endotoxin could have obscured significant analyte production in antigen stimulated samples. We have previously also observed the same pattern of higher unstimulated than stimulated analyte levels in samples that were collected directly into QFT tubes (unpublished data). Future studies should employ collection directly into QFT tubes.

The main limitation of our study is the relatively small number of study participants and the cross – sectional design. Longitudinal cohort studies will be required with careful clinical characterization of participants into TB infection and disease groups to validate the accuracies and the cut-off values of the markers identified in this study. This will require a prospective study whereby misclassification of active and latent TB by these cytokine combinations is noted. Future studies should also access the utility of the three-marker tests in smear negative TB, extrapulmonary TB, immune compromised subjects (especially HIV infected patients), children and people with other lung infections like acute bacterial pneumonia. Additional biomarkers should also be evaluated as new multiplex assays become available. Additionally, development of suitable point-of-care tests will be needed, using easy-to-use, readily accessible and less costly techniques like ELISA assays (as opposed to Luminex).

## Conclusion

In conclusion, our preliminary results suggest that active TB may be accurately identified within 24 hours utilizing an adaptation of the commercial QFT assay where detection of a combination of three host markers (selected from EGF, sCD40L, MIP-1β, VEGF, TGF-α or IL-1α) is performed on QFT supernatants. The results hold promise for the development of a rapid and sensitive test for active TB.

## Competing interests

The authors declare that they have no competing interests.

## Authors' contributions

NC and GW conceived and designed the study, wrote the draft of the manuscript and interpreted the data after statistical analysis. NC performed and interpreted all the laboratory assays and participated in the analysis of the data. GB and PVH participated in the recruitment of participants, writing up and editing of the manuscript. MK participated in the analysis and interpretation of the data and writing up of the manuscript. All authors read and approved the final manuscript.

## Pre-publication history

The pre-publication history for this paper can be accessed here:



## Supplementary Material

Additional file 1 **Abilities of combinations of analytes in GDA models to discriminate between positive QFT results**. The data provides information on the performances of combinations of markers in general discriminant analysis (GDA) models in discriminating between positive QFT results as either pulmonary TB or LTBI. TB = pulmonary TB, LTBI = household contact with a positive QFT and TST result. Click here for file

Additional file 2 **Abilities of combinations of analytes in SVM models to discriminate between positive QFT results**. The data provides information on the performances of combinations of markers in support vector machine (SVM) models in discriminating between positive QFT results as either pulmonary TB or LTBI. TB = pulmonary TB, LTBI = household contact with a positive QFT and TST result. Click here for file

Additional file 3 **Abilities of combinations of analytes in GDA models to discriminate between all TB cases and household contacts**. The data provides information on the ability of combinations of analytes in general discriminant analysis (GDA) models to discriminate between pulmonary TB cases and household contacts, irrespective of QFT results. Click here for file

Additional file 4 **Abilities of combinations of analytes in SVM models to discriminate between all TB cases and household contacts**. The data provides information on the ability of combinations of analytes in support vector machine (SVM) models to discriminate between pulmonary TB cases and household contacts, irrespective of QFT results. Click here for file

## References

[B1] Pai M, Riley LW, Colford JM (2004). Interferon-gamma assays in the immunodiagnosis of tuberculosis: a systematic review. Lancet Infect Dis.

[B2] Pai M, Kalantri S, Dheda K (2006). New tools and emerging technologies for the diagnosis of tuberculosis: part I. Latent tuberculosis. Expert Rev Mol Diagn.

[B3] Menzies D, Pai M, Comstock G (2007). Meta-analysis: new tests for the diagnosis of latent tuberculosis infection: areas of uncertainty and recommendations for research. Ann Intern Med.

[B4] Mandalakas AM, Hesseling AC, Chegou NN, Kirchner HL, Zhu X, Marais BJ, Black GF, Beyers N, Walzl G (2008). High level of discordant IGRA results in HIV-infected adults and children. Int J Tuberc Lung Dis.

[B5] Connell TG, Rangaka MX, Curtis N, Wilkinson RJ (2006). QuantiFERON-TB Gold: state of the art for the diagnosis of tuberculosis infection?. Expert Rev Mol Diagn.

[B6] Richeldi L, Ewer K, Losi M, Roversi P, Fabbri LM, Lalvani A (2006). Repeated tuberculin testing does not induce false positive ELISPOT results. Thorax.

[B7] Hanna BA, Ebrahimzadeh A, Elliott LB, Morgan MA, Novak SM, Rusch-Gerdes S, Acio M, Dunbar DF, Holmes TM, Rexer CH, Savthyakumar C, Vannier AM (1999). Multicenter evaluation of the BACTEC MGIT 960 system for recovery of mycobacteria. J Clin Microbiol.

[B8] Trajman A, Pai M, Dheda K, van Zyl SR, Zwerling AA, Joshi R, Kalantri S, Daley P, Menzies D (2008). Novel tests for diagnosing tuberculous pleural effusion: what works and what does not?. Eur Respir J.

[B9] WHO REPORT (2008). Global Tuberculosis Control surveillance, planning, financing.

[B10] QuantiFERON TB Gold (In Tube method) package insert. http://www.cellestis.com/IRM/Company/ShowPage.aspx?CPID=1170.

[B11] Human Cytokine/Chemokine Panel – 29 Plex. http://www.millipore.com/catalogue/item/MPXHCYTO-60K-29#.

[B12] Desem N, Jones SL (1998). Development of a human gamma interferon enzyme immunoassay and comparison with tuberculin skin testing for detection of Mycobacterium tuberculosis infection. Clin Diagn Lab Immunol.

[B13] Djoba Siawaya JF, Bapela NB, Ronacher K, Veenstra H, Kidd M, Gie R, Beyers N, van Helden P, Walzl G (2008). Immune parameters as markers of tuberculosis extent of disease and early prediction of anti-tuberculosis chemotherapy response. J Infect.

[B14] Bermudez LE, Petrofsky M, Shelton K (1996). Epidermal growth factor-binding protein in Mycobacterium avium and Mycobacterium tuberculosis: a possible role in the mechanism of infection. Infect Immun.

[B15] Parker AE, Bermudez LE (2000). Sequence and characterization of the glyceraldehyde-3-phosphate dehydrogenase of Mycobacterium avium: correlation with an epidermal growth factor binding protein. Microb Pathog.

[B16] Husain N, Awasthi S, Haris M, Gupta RK, Husain M (2008). Vascular endothelial growth factor as a marker of disease activity in neurotuberculosis. J Infect.

[B17] Kiropoulos TS, Kostikas K, Gourgoulianis KI (2005). Vascular endothelial growth factor levels in pleural fluid and serum of patients with tuberculous pleural effusions. Chest.

[B18] Alatas F, Alatas O, Metintas M, Ozarslan A, Erginel S, Yildirim H (2004). Vascular endothelial growth factor levels in active pulmonary tuberculosis. Chest.

[B19] Fahey TJ, Tracey KJ, Tekamp-Olson P, Cousens LS, Jones WG, Shires GT, Cerami A, Sherry B (1992). Macrophage inflammatory protein 1 modulates macrophage function. J Immunol.

[B20] Sherry B, Espinoza M, Manogue KR, Cerami A (1998). Induction of the chemokine beta peptides, MIP-1 alpha and MIP-1 beta, by lipopolysaccharide is differentially regulated by immunomodulatory cytokines gamma-IFN, IL-10, IL-4, and TGF-beta. Mol Med.

[B21] Saltini C, Colizzi V (1999). Soluble immunological markers of disease activity in tuberculosis. Eur Respir J.

[B22] Hernandez-Pando R, Orozco H, Arriaga K, Sampieri A, Larriva-Sahd J, Madrid-Marina V (1997). Analysis of the local kinetics and localization of interleukin-1 alpha, tumour necrosis factor-alpha and transforming growth factor-beta, during the course of experimental pulmonary tuberculosis. Immunology.

[B23] Schoenberger SP, Toes RE, van dV, Offringa R, Melief CJ (1998). T-cell help for cytotoxic T lymphocytes is mediated by CD40-CD40L interactions. Nature.

[B24] Mizusawa M, Kawamura M, Takamori M, Kashiyama T, Fujita A, Usuzawa M, Saitoh H, Ashino Y, Yano I, Hattori T (2008). Increased synthesis of anti-tuberculous glycolipid immunoglobulin G (IgG) and IgA with cavity formation in patients with pulmonary tuberculosis. Clin Vaccine Immunol.

[B25] Kobashi Y, Mouri K, Obase Y, Fukuda M, Miyashita N, Oka M (2007). Clinical evaluation of QuantiFERON TB-2G test for immunocompromised patients. Eur Respir J.

[B26] Ferrara G, Losi M, D'Amico R, Roversi P, Piro R, Meacci M, Meccugni B, Dori IM, Andreani A, Bergamini BM, Mussini C, Rumpianesi F, Fabbri LM, Richeldi L (2006). Use in routine clinical practice of two commercial blood tests for diagnosis of infection with Mycobacterium tuberculosis: a prospective study. Lancet.

[B27] Ruhwald M, Bjerregaard-Andersen M, Rabna P, Kofoed K, Eugen-Olsen J, Ravn P (2007). CXCL10/IP-10 release is induced by incubation of whole blood from tuberculosis patients with ESAT-6, CFP10 and TB7.7. Microbes Infect.

[B28] Ruhwald M, Petersen J, Kofoed K, Nakoaka H, Cuevas LE, Lawson L, Squire SB, Eugen-Olsen J, Ravn P (2008). Improving T-Cell Assays for the Diagnosis of Latent TB Infection: Potential of a diagnostic Test Based on IP-10. Plos One.

[B29] Ruhwald M, Bodmer T, Maier C, Jepsen M, Haaland MB, Eugen-Olsen J, Ravn P (2008). Evaluating the potential of IP-10 and MCP-2 as biomarkers for the diagnosis of tuberculosis. Eur Respir J.

